# Are There Ethnic Inequalities in Revascularisation Procedure Rate after an ST-Elevation Myocardial Infarction?

**DOI:** 10.1371/journal.pone.0136415

**Published:** 2015-09-14

**Authors:** Aloysia A. M. van Oeffelen, Saskia Rittersma, Ilonca Vaartjes, Karien Stronks, Michiel L. Bots, Charles Agyemang

**Affiliations:** 1 Julius Center for Health Sciences and Primary Care, University Medical Center Utrecht, 3508 GA, Utrecht, the Netherlands; 2 Department of Cardiology, University Medical Center Utrecht, 3508 GA, Utrecht, The Netherlands; 3 Department of Public Health, Academic Medical Center, University of Amsterdam, 1100 DD, Amsterdam, The Netherlands; University of Bologna, ITALY

## Abstract

**Background:**

Previously, ethnic inequalities in prognosis after a first acute myocardial infarction were observed in the Netherlands. This might be due to differences in revascularisation rate between ethnic minority groups and ethnic Dutch. Therefore, we investigated inequalities in revascularisation rate after occurrence of an ST-elevation myocardial infarction (STEMI) between first generation ethnic minority groups (henceforth, migrants) and ethnic Dutch.

**Methods:**

All STEMI events between 2006 and 2011 were identified in a subset of the Achmea Health Database, which records medical care to persons insured at the Achmea health insurance company, a major health insurance company in the central part of the Netherlands. Ethnic Dutch and migrants from Suriname (Hindustani Surinamese and non-Hindustani Surinamese), Morocco, and Turkey were included (n = 1,765). Multivariable Cox proportional hazards regression analyses were used to identify ethnic inequalities in revascularisation rate (percutaneous coronary intervention (PCI) and coronary artery bypass grafting (CABG)) after a STEMI event.

**Results:**

On average, 73.2% of STEMI events were followed by a revascularisation procedure. After adjustment for confounders (age, sex, degree of urbanization) no significant differences in revascularisation rate were found between the ethnic Dutch population and Hindustani Surinamese (HR: 1.04; 0.85–1.27), non-Hindustani Surinamese (HR: 0.98; 0.63–1.51), Moroccan (HR: 0.94; 0.77–1.14), and Turkish migrants (HR: 1.04; 0.88–1.24). Additional adjustment for comorbidity and neighborhood income did not change our findings.

**Conclusion:**

Our study suggests no ethnic inequalities in revascularisation rate after a STEMI event. This finding is in agreement with the universally accessible health care system in the Netherlands.

## Introduction

An ST-elevation myocardial infarction (STEMI) is characterised by an acute total occlusion of one or more coronary arteries, resulting in necrosis of the myocardium. In case of a STEMI, as diagnosed on the ECG, treatment is immediately indicated. The occlusion can be resolved using medication therapy (e.g. fibrinolysis) or a revascularisation procedure, such as percutaneous coronary intervention (PCI) and coronary artery bypass grafting (CABG). In the past decade, more evidence became available emphasizing the beneficial effect of revascularisation procedures in STEMI patients.[1,2] Recent guidelines therefore recommend such a procedure over medication therapy after a STEMI event, as it improves prognosis when performed within an adequately short time frame (preferably within 90 minutes after first medical contact).[3–5] Guidelines do not mention contra-indications for performing a revascularisation procedure. However, in 2004 the European Heart Survey reported that only 61% of all STEMI patients received a revascularisation procedure, suggesting a marked underuse.[6]

A recent study of our group showed that mortality after a first hospitalization for an acute myocardial infarction (AMI) was higher among ethnic minority groups compared with the ethnic Dutch population.[7] We hypothesized that ethnic inequalities in cardiac revascularisation procedures might partially explain these findings, since this was also observed previously in USA studies. African Americans were less likely to receive a revascularisation procedure after an acute myocardial infarction (AMI) than their White American counterparts, which explained their higher mortality into some extent.[8–11] An important underlying factor was the lack of health insurance and the inability to pay for such an expensive procedure, especially among the African-American population.[12] In Europe, literature concerning ethnic inequalities in cardiac revascularisation procedures is scarce. Many European countries have a health care system with universal access to acute in-hospital care which aims at minimizing health care inequalities. However, in the United Kingdom (where revascularisation procedures are also performed free of charge for patients) a lower revascularisation procedure rate among African and South-Asian minorities compared with the general population was reported, even after adjustment for actual need.[13–15] Yet, more recent data do not show ethnic inequalities in revascularisation procedure rate in the UK anymore.[16] Evidence in other European countries, such as the Netherlands, is lacking. Given the absence of information on this issue, and the possibility that differences in revascularisation rate between ethnic groups may explain ethnic inequalities in prognosis after AMI, we set out to study differences in revascularisation procedure rate (PCI and CABG) after a STEMI event between first generation ethnic minority groups (henceforth, migrants) and ethnic Dutch in the Netherlands.

## Methods

### Data sources

Data were extracted from the Achmea Health Database (AHD). In the Netherlands all inhabitants are by law obliged to have medical insurance coverage. The Achmea health insurance company is the main health insurance company in the central part of the Netherlands as it provides health care coverage for more than one million Dutch residents, of which 20% belongs to an ethnic minority group. The AHD records payments for the provision of all medical care to insured patients. Although the AHD is not completely representative for the entire Dutch population, it does represent the urbanised areas of the Netherlands. Detailed information about the AHD is described previously.[17] Because of a transition in type of registration procedure in 2005, our dataset was restricted to the period 2006 to 2011. Due to privacy issues we only received a subset of the AHD. The subset consisted of data from all insured Hindustani Surinamese, Moroccan, and Turkish ethnic minority groups of ≥30 years of age (n = 162,484), whose ethnicity was based on nationality and surnames, as described previously.[18] In short, first generation ethnic minorities were selected by their nationality. To select the second and third generation, the surnames of the selected first generation ethnic minorities were matched with the remaining of the database and visually controlled on origin of the name. Furthermore, our subset consisted of a representative sample of the remaining insured group of ≥30 years of age (n = 194,993), encompassing ethnic Dutch and ethnic minorities other than Hindustani Surinamese, Moroccan, and Turkish. This resulted in a cohort of 357,477 insured persons. Approval from the AHD research committee was obtained prior to accessing the data. From the AHD cohort, all inpatient and outpatient STEMI events were selected. Subsequently, for every patient only the first event within 30 days was retained. For each patient we determined whether they received a PCI or CABG procedure. They were followed and censored in case of death, end of Achmea insurance, or the end of the study period at 31 December 2011, whichever came first. The dataset was linked with the Population Register (PR), Hospital Discharge Register (HDR), and the Regional Income Survey (RIS) to obtain information regarding country of birth, comorbidity, and neighborhood income (as indicator for socioeconomic status).

### Determinants

#### Ethnic background

Migrant groups were identified using the country of birth and the country of birth of the parents, as recorded in the PR. A patient belonged to a migrant group if he/she was born abroad and at least one of the parents was born abroad.[19] Migrants from Suriname, Morocco, and Turkey were selected. Other migrant groups were excluded due to small numbers. The Surinamese population in the Netherlands is ethnically diverse, and mainly consists of people from Hindustani South-Asian descent and from West-African descent. We disaggregated the Hindustani Surinamese from the non-Hindustani Surinamese by using Hindustani surnames available from the Achmea Health Database and the Dutch SUNSET study.[18,20] A patient was considered ethnic Dutch when both parents were born in the Netherlands. The final cohort comprised 1,765 STEMI events.

#### Degree of urbanization

The degree of urbanisation was based on the population density of the city where the insured person lived on 1 January 2006, extracted from the AHD. Five categories were constructed: very rural, rural, rural/urban, urban, and very urban.

#### Neighborhood socioeconomic status

Socioeconomic status (SES) was based on income data registered in the RIS.[21] The RIS started in 1994, when a representative sample of 1.9 million Dutch citizens was selected. Every year, the sample was corrected for emigration and mortality on one hand, and immigration and birth on the other hand. For all persons belonging to the households of the sample population (about one third of the Dutch population) the household income was available. Within each neighborhood, the mean of all registered household incomes was calculated and subsequently assigned to all residents living in that neighborhood. For this study, the neighborhood income of the patients during 2006 was assigned. Subsequently, neighborhood income was divided into SES tertiles, with the first tertile representing the lowest income group.

#### Comorbidity

Presence and extent of comorbidity were determined with the Charlson index score [22], based on discharge diagnosis in the Hospital Discharge Register from 1995 to the date of the STEMI event. The Charlson index ranges from zero to six (cut-off value), with zero representing no comorbidity. It proved to be a reliable and valid method to measure co-morbidity in clinical research.[23]

### Data analysis

Patient characteristics were analysed within the ethnic Dutch population and the migrant groups separately, using cross tables and frequency tables. Cox proportional hazard regression analyses were used to calculate the difference in revascularisation rate after the STEMI event between migrant groups and ethnic Dutch (reference). Three consecutive models were built. Model one included only confounders (age, sex, degree of urbanisation). Model two and three also included comorbidity and neighborhood SES, to investigate whether these factors could explain found relations. We used SPSS software, version 20.0 (SPSS Inc, Chicago, Illinois, USA). All analyses were performed in accordance with privacy legislation Netherlands.

### Ethics statement

No separate ethical approval was necessary for the use of the Achmea health database data. All data were analyzed anonymously.

## Results

Table 1 presents the patient characteristics of the study population, comprising 1,765 STEMI events, of which 36% belonged to an ethnic minority group. Within the ethnic Dutch population, 71.0% of STEMI events were followed by a revascularisation procedure. Within the migrant groups this percentage was higher (ranging from 71.9% among Moroccans to 80.6% among Turkish migrants). Migrants were more often men, younger, and lived in more urbanised low SES neighborhoods. Comorbidity was about equal between migrants and ethnic Dutch, except for Moroccans who had substantially less comorbidity. Within all groups, PCI was the most commonly performed revascularisation procedure.

**Table 1:** Characteristics of persons with a STEMI event ≥30 years of age in the Achmea Health Database between 2006 and 2011.

	Ethnic Dutch	Total Surinamese	Hindustani Surinamese	Non-Hindustani Surinamese	Moroccan	Turkish	Total
**STEMI** * **events**	1,137	199	171	28	192	237	1,765
**Person-days at risk**	248,199	44,552	37,009	7,543	57,413	45,675	395,839
**Procedures %**	71.0	78.4	78.4	78.6	71.9	80.6	73.2
**Procedure type %**							
PCI †	67.0	74.4	74.3	75.0	69.8	78.9	69.7
CABG ‡	6.9	9.0	9.4	-**	6.2	7.6	7.2
**Median age in years (IQR** x**)**	69 (57–79)	56 (48–65)	56 (48–65)	56 (45–66)	62 (52–70)	54 (47–65)	64 (53–75)
**Men %**	59.5	74.4	74.3	75.0	83.9	81.0	66.7
**Neighborhood SES %** §							
Tertile 1(lowest income)	33.9	57.8	55.6	71.4	71.9	72.2	45.8
Tertile 2 (medium income)	32.6	27.6	29.2	-**	14.6	18.1	28.2
Tertile 3 (highest income)	33.5	14.6	15.2	-**	13.5	9.7	26.0
**Degree of urbanisation %** ll							
Very urban	28.3	75.9	76.0	75.0	77.6	58.2	43.1
Urban	21.3	10.6	9.9	-**	10.9	18.1	18.5
Urban/rural	21.3	13.1	13.5	-**	9.4	15.6	18.3
Rural	21.5	-**	-**	-**	-**	7.6	15.1
Very rural	7.6	-**	-**	-**	-**	-**	5.0
**Charlson index > 0%** #	69.6	69.8	69.6	71.4	23.4	64.6	63.9

* ST-elevation myocardial infarction

^†^ Percutaneous coronary intervention

^‡^ Coronary artery bypass grafting

x Interquartile range

^§^ Socioeconomic status

^ll^ Based on population density (number of residents per km^2^). Very urban = >2000, urban = 1001–2000, urban/rural = 501–1000, rural = 251–500, very rural = <251

^#^ At least one hospitalisation for a diagnosis included in the Charlson comorbidity index from 1995 until the STEMI event

** Not given in line with the Dutch data protection guideline as the number of cases was less than ten

After adjustment for the confounders age, sex, and degree of urbanization (model 1) there were no significant differences in revascularisation procedure rate between migrant groups and ethnic Dutch (Table 2). Adding comorbidity (model 2) and neighborhood SES (model 3) did not markedly influence results. There were also no differences in revascularisation procedure rate between migrant groups and ethnic Dutch after stratification for age and sex (results not shown).

**Table 2:** Difference in revascularisation procedure rate after a STEMI event between migrants and the ethnic Dutch population ≥30 years of age (HR (95% CI))*.

	Procedure (%)	Model 1 †	Model 2‡	Model 3x
**Ethnic Dutch**	807 (71.0)	1.00	1.00	1.00
**Total Surinamese**	156 (78.4)	1.04 (0.86–1.25)	1.04 (0.86–1.26)	1.03 (0.85–1.24)
Hindustani Surinamese	134 (78.4)	1.04 (0.85–1.27)	1.04 (0.85–1.27)	1.03 (0.84–1.26)
Non-Hindustani Surinamese	22 (78.6)	0.98 (0.63–1.51)	0.98 (0.64–1.52)	0.97 (0.62–1.49)
**Moroccan**	138 (71.9)	0.94 (0.77–1.14)	0.89 (0.73–1.09)	0.87 (0.71–1.07)
**Turkish**	191 (80.6)	1.04 (0.88–1.24)	1.04 (0.87–1.23)	1.01 (0.85–1.21)

^†^ Adjusted for age, sex, and degree of urbanisation

^‡^ Adjusted for age, sex, degree of urbanisation, and Charlson comorbidity index

x Adjusted for age, sex, degree of urbanisation, Charlson comorbidity index, and neighborhood SES

## Discussion

Our study shows no ethnic inequalities in revascularisation rate after an ST-elevation myocardial infarction in the Netherlands.

### Discussion of main findings

Our results are in contrast with the overwhelming literature from the USA, showing considerable lower revascularisation rates among African Americans compared with White Americans after a coronary event.[8,10,11] A major difference between the USA and the Netherlands is the universal access to health care implemented in Dutch society, in which everyone is obliged by law to have health insurance. In the Netherlands, persons with a low income are being supported by the government through health care benefits, and PCI and CABG are available without additional costs. In the United States, still more than 16% of the population is uninsured. This percentage is higher in African Americans (20.8%) than in non-Hispanic Whites (11.7%).[12] The inability of the uninsured to pay for PCI and CABG procedures may partially underlie the lower procedure rate in African Americans. Nevertheless, lack of health insurance cannot fully explain the ethnic inequalities, since Cram et al. recently reported a lower revascularisation rate after AMI among African Americans independent of health insurance.[24] Also a study that was executed in Medicare beneficiaries only, still revealed lower revascularisation rates among African Americans.[8] However, in most studies executed in the USA, not only STEMI events were included but also non-ST-elevation myocardial infarctions (non-STEMI). The choice to perform a revascularisation procedure after a non-STEMI is much more dependent on clinical symptoms than after a STEMI.[25] The underlying indication, based on clinical symptoms, may differ across ethnic groups. Indeed, some studies reported that the lower revascularisation procedure rate among African Americans was partially explained by a lower coronary artery disease burden and severity.[26–28] Yet, one study which included African Americans and Whites with a similar disease severity still showed a lower revascularisation rate among African Americans.[10] More reluctance in African Americans than in Whites to accept a recommendation for surgery or PCI may additionally underlie the lower procedure use.[29] The absent ethnic inequalities in revascularisation procedure rate in our study not only suggest equal provision of care by physicians, but also equal uptake of care by STEMI patients in the Netherlands.

Until now, European studies on ethnic inequalities in revascularisation rate after a coronary event were limited to the UK.[13–15] Just as in the Netherlands, UK patients have free access to acute in-hospital care. However, studies executed in the 2000s still found lower rates of revascularisation procedures after a coronary event among African and South-Asian minorities. A major drawback in two of these studies was that they did not adjust for actual need for a revascularisation procedure. This is an important factor in the decision to perform a revascularisation procedure after a non-ST-elevation cardiac event.[4] Although one study also found ethnic inequalities among those deemed appropriate for revascularisation, this study dates back from 2002 and is therefore considered outdated given the current guidelines.[13] The most recent UK study from 2013 did not find ethnic inequalities in revascularisation rate after AMI anymore.[16]

Recently, our group reported a higher mortality rate after a first hospitalisation for AMI among migrant groups compared with the ethnic Dutch population.[7] Results from our present study suggest that this cannot be explained by ethnic differences in revascularisation procedure rate. However, we did not have any information regarding the time span between symptom onset and the revascularisation procedure. International literature suggests that this time span might be longer among ethnic minority groups than among the ‘majority population’.[30] Since ‘time is muscle’ a longer time delay can substantially undermine the beneficial effect of a revascularisation procedure on prognosis.[31] Ethnic inequalities in the time period between onset of symptoms and the revascularisation procedure has to be investigated in future research.

### Considerations

Ethnic inequalities in revascularisation rate after AMI is rarely studied in countries with universal access to health care. We had the opportunity to use data from the Achmea health database, in which registration of procedures is extensively controlled for the reason of financial reimbursement.[31] We used surname algorithms in combination with (parental) country of origin to disaggregate the Hindustani Surinamese from other Surinamese, who have very different cardiovascular disease profiles. Furthermore, our analyses were based on those with a STEMI event only. The choice to perform a revascularisation procedure after a STEMI is more straightforward than after a non-STEMI, and not dependent on other clinical factors (such as coronary artery disease burden and severity). The absence of ethnic inequalities in revascularisation procedure rate after STEMI as reported in this study therefore truly reflects equity in acute in-hospital health care use for individuals with STEMI.

Our study has some limitations. Firtst, although registration of procedures in the Achmea health database is extensively controlled, this is a very rough check to secure that the registered procedures are in agreement with the registered diagnosis. There are no detailed validation studies available that have investigated the sensitivity and the positive predictive value of registered diagnosis and procedures. Second, the revascularisation rate of 73% seems rather low, since there are no clear contra-indications to perform a revascularisation procedure among STEMI patients. However, this percentage is higher as the previously reported 61% in Europe.[6] More research is necessary to understand the underlying factors of this low percentage. Since we do not assume these factors to differ between ethnic groups, we believe it would not have affected our results concerning ethnic inequalities. Third, in the Achmea health database, persons living in urban areas of the Netherlands are overrepresented. However, correction for degree of urbanization did not change relations, which indicates that results are representative for the general Dutch population. Fourth, we did not investigate the difference in prognosis after a STEMI between ethnic groups, and the effect of a revascularisation procedure on prognosis. This would be interesting for future research.

### Conclusion

Our findings show no ethnic inequalities in revascularisation procedure rate after an ST-elevation myocardial infarction in the Netherlands, suggesting equity in acute in-hospital care. This is in agreement with the universally accessible health care system implemented in Dutch society. The previously observed higher mortality rate after AMI among ethnic minority groups in the Netherlands are therefore unlikely explained by differences in revascularisation procedure rate.
